# Astroglial-mediated remodeling of the interhemispheric midline during telencephalic development is exclusive to eutherian mammals

**DOI:** 10.1186/s13064-017-0086-1

**Published:** 2017-05-30

**Authors:** Ilan Gobius, Rodrigo Suárez, Laura Morcom, Annalisa Paolino, Timothy J. Edwards, Peter Kozulin, Linda J. Richards

**Affiliations:** 1 Queensland Brain Institute, The University of Queensland, St Lucia, Brisbane, 4072 Australia; 2 Faculty of Medicine, The University of Queensland, Herston, Brisbane, 4006 Australia; 3 School of Biomedical Sciences, The University of Queensland, St Lucia, Brisbane, 4072 Australia

**Keywords:** Interhemispheric remodeling, Interhemispheric fissure, Corpus callosum, Telencephalic commissure formation

## Abstract

The corpus callosum forms the major interhemispheric connection in the human brain and is unique to eutherian (or placental) mammals. The developmental events associated with the evolutionary emergence of this structure, however, remain poorly understood. A key step in callosal formation is the prior remodeling of the interhemispheric fissure by embryonic astroglial cells, which then subsequently act as a permissive substrate for callosal axons, enabling them to cross the interhemispheric midline. However, whether astroglial-mediated interhemispheric remodeling is unique to eutherian mammals, and thus possibly associated with the phylogenetic origin of the corpus callosum, or instead is a general feature of mammalian brain development, is not yet known. To investigate this, we performed a comparative analysis of interhemispheric remodeling in eutherian and non-eutherian mammals, whose lineages branched off before the evolution of the corpus callosum. Whole brain MRI analyses revealed that the interhemispheric fissure is retained into adulthood in marsupials and monotremes, in contrast to eutherians (mice), in which the fissure is significantly remodeled throughout development. Histological analyses further demonstrated that, while midline astroglia are present in developing marsupials, these cells do not intercalate with one another through the intervening interhemispheric fissure, as they do in developing mice. Thus, developing marsupials do not undergo astroglial-mediated interhemispheric remodeling. As remodeling of the interhemispheric fissure is essential for the subsequent formation of the corpus callosum in eutherians, our data highlight the role of astroglial-mediated interhemispheric remodeling in the evolutionary origin of the corpus callosum.

## Introduction

Interhemispheric brain connections are crucial for the integration of neural processes between the left and right cerebral hemispheres. In eutherian (or placental) mammals, such as humans and mice, three commissures connect the two telencephalic hemispheres: the anterior commissure, the hippocampal commissure and the corpus callosum [1–3]. In the human brain, the corpus callosum forms the major interhemispheric connection between the two cerebral hemispheres [4]. This structure is comprised of approximately 200 million axons [5], and integrates sensory, motor and associative processes between the hemispheres [6, 7]. While non-eutherian mammals, such as marsupials and monotremes, have an anterior commissure and a hippocampal commissure, the corpus callosum is found exclusively in eutherians. However, the events that led to the emergence of the corpus callosum in eutherian ancestors are still poorly understood [1–3].

During eutherian brain development, the callosal tract is predominantly formed by long-range projection neurons in layers 2/3 and 5 of the neocortex that extend their axons medially across the interhemispheric midline through a region that is formerly separated by the interhemispheric fissure (IHF) [8–11]. In contrast, interhemispheric neocortical projections in marsupial and monotreme brains cross the midline more ventrally, via the evolutionarily-older anterior commissure [2, 3].

The IHF is predominantly comprised of leptomeningeal fibroblasts and extracellular matrix, which together form a non-permissive barrier for callosal axons during development [8, 12]. Recently, we demonstrated that prior remodeling of the IHF by specialized astroglial cells is essential for callosal tract formation, and that aberrant retention of the IHF due to defects in this process results in agenesis (or absence) of the corpus callosum in both mice and humans [8]. Interhemispheric remodeling is initiated by the transition of these specialized glial cells (known as midline zipper glia; MZG) from radial glia into multipolar astroglia. This transition into a multipolar state is required for MZG cells to intercalate with one another across the IHF and degrade the intervening leptomeninges. Callosal axons then use intercalated MZG cells as a substrate to cross the interhemispheric midline [8]. As astroglial-mediated interhemispheric remodeling is a developmental process that is critical for the formation of the corpus callosum, we asked whether this cellular process is exclusive to eutherian mammals, and therefore associated with the phylogenetic origin of the corpus callosum, or instead is a more general mammalian process that can also be observed in non-eutherian species.

## Results

**Fig. 1:**
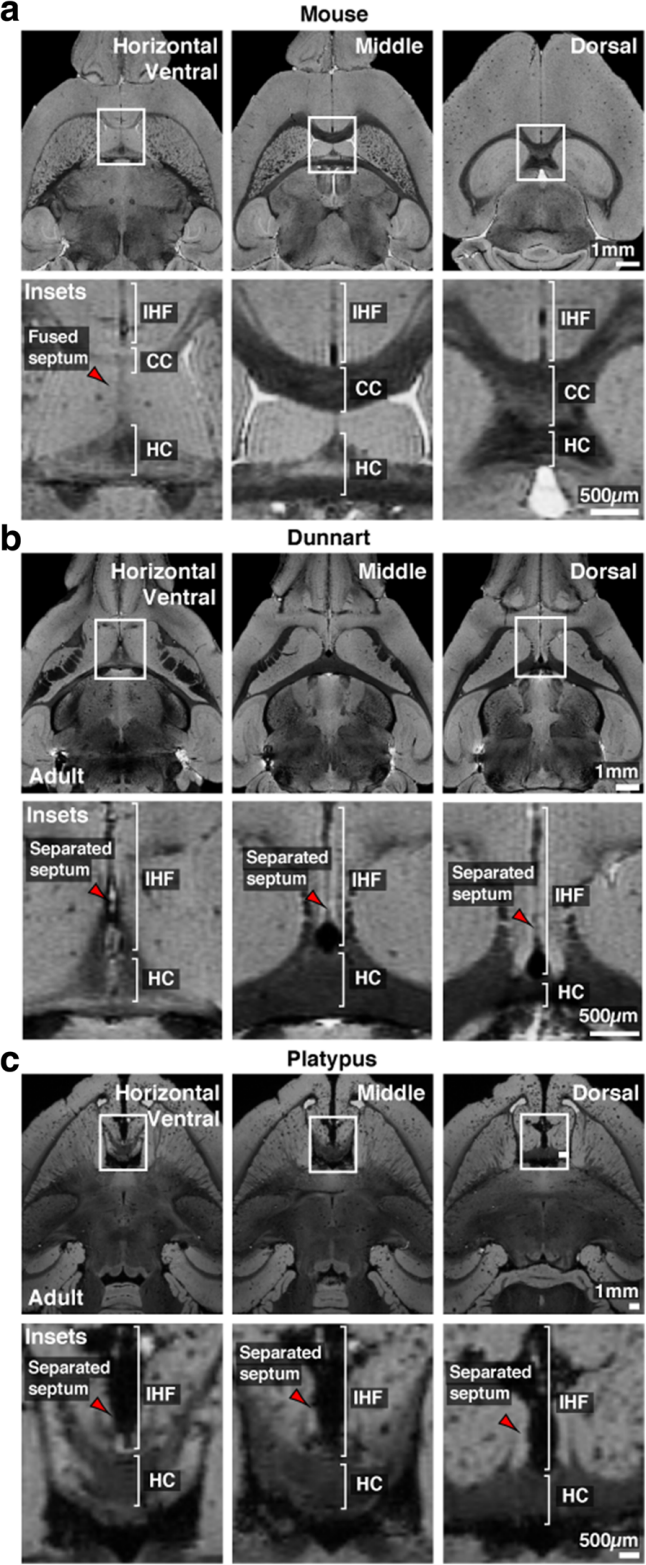
The IHF is retained into adulthood in both marsupials and monotremes. Comparison of horizontal T1-weighted structural MRI images from adult mouse (**a**), dunnart (**b**) and platypus (**c**) brains. Brackets indicate the distribution of the IHF, hippocampal commissure (HC) and corpus callosum (CC) along the midline. Note separation of the septum in dunnart and platypus brains, compared to the fused septum in mouse brains (*red arrowheads*)

To begin to address this question, we first investigated whether interhemispheric remodeling occurs in mammalian lineages that branched off before the evolution of the corpus callosum, such as marsupials and monotremes [3]. Structural T1-weighted MRI images of the interhemispheric midline from fixed brains of adult mice (*Mus musculus*, Placentalia; Fig. 1a) were compared with equivalent images obtained from fixed brains of adult fat-tailed dunnarts (*Sminthopsis crassicaudata*, Marsupialia, Fig. 1b) and platypus (*Ornithorhynchus anatinus*, Monotremata; Fig. 1c). In adult mice, the IHF is significantly remodeled (Fig. 1a), such that the IHF only occupies approximately 60% of the length of the midline [8]. In dunnarts and platypus, however, we observed that the majority of the IHF appears to be retained into adulthood, despite the presence of a large hippocampal commissure in both of these species (Fig. 1b and c). Notably, retention of the IHF in dunnarts and platypus results in separation of the septal halves (red arrowheads; Fig. 1b and c), unlike the mouse septum, which is fused along its midline (red arrowhead; Fig. 1a). Together, these observations suggest that interhemispheric remodeling may not occur in non-eutherian mammals.

In order to further confirm the absence of interhemispheric remodeling in non-eutherian mammals, we then compared both mice and dunnarts at early embryonic/postnatal and adult stages to ascertain whether interhemispheric remodeling occurs during dunnart development, as it does in mice. These marsupials are born with a highly underdeveloped brain that is approximately equivalent to the developing mouse brain at embryonic day (E)10 [13]. Thus, the majority of dunnart telencephalic development occurs postnatally while the animal is attached to the teat in the mother’s pouch.

**Fig. 2:**
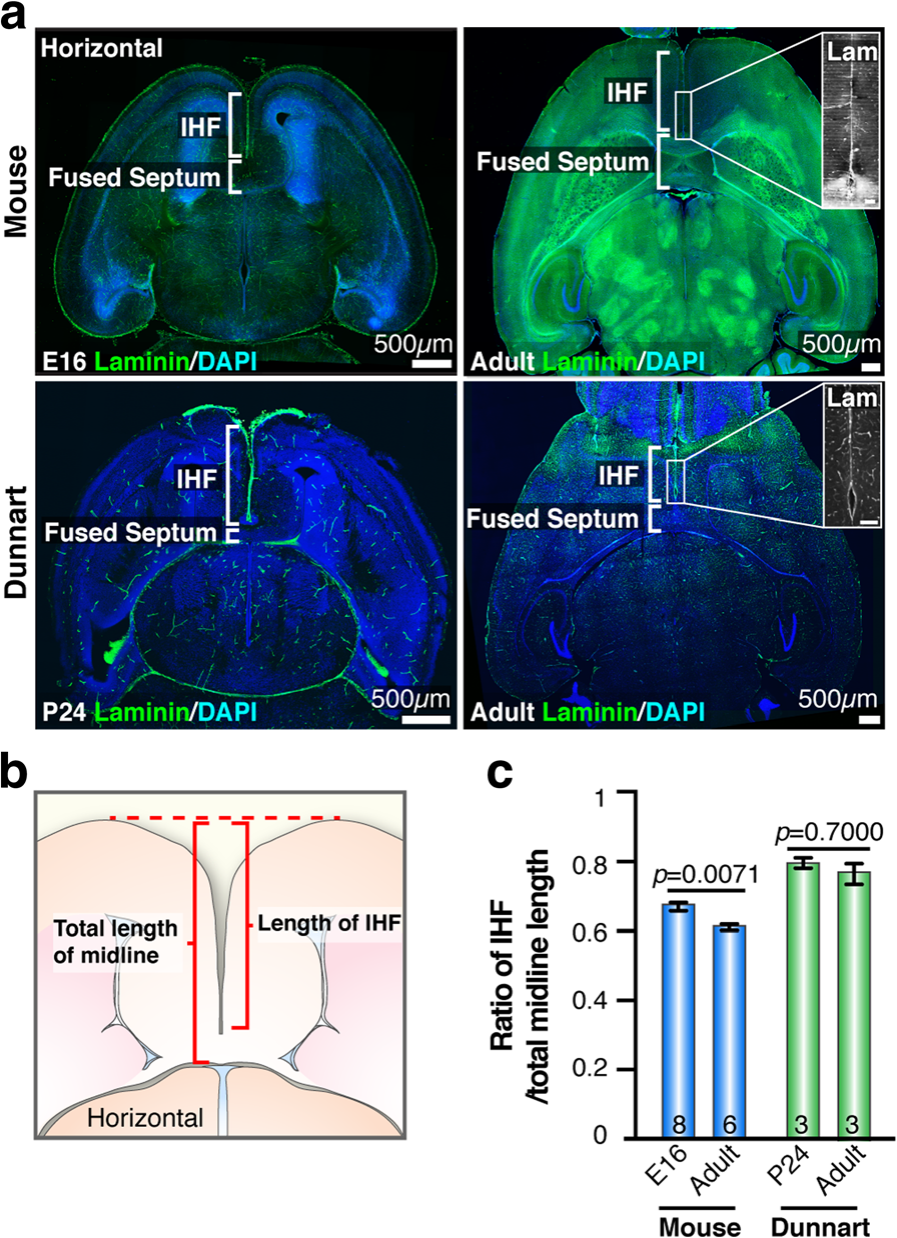
Developmental remodeling of the IHF does not occur in marsupials. **a** Immunofluorescence for Laminin (*green*), counterstained with DAPI (*blue*) shows the length of the IHF and the extent of fused septum (brackets) in horizontal sections of the forebrain from E16 and adult mice (*upper* panels), as well as P24 and adult dunnarts (*lower* panels). Insets in right-hand panels show Laminin expression (*white*) at adult stages within the IHF. **b** Schematic of midline and IHF length measurements taken to quantify normalized IHF length. **c** Quantification of normalized IHF length in E16 and adult mouse brains, compared with normalized IHF length in dunnarts at the equivalent stages (P24 and adult). Data are represented as means ± SEM (n-values within bars). Scale bars within insets = 100 μm

To assess the length of the IHF throughout development, we immunolabeled the basement membrane and leptomeningeal fibroblasts that form the IHF with pan-Laminin and stained cell nuclei with 4′,6-diamidino-2-phenylindole, dihydrochloride (DAPI) at E16 in mice, postnatal day (P)24 in dunnarts, as well as adult stages for both species (Fig 2a). Notably, we observed that pan-Laminin predominantly labels the leptomeninges and vasculature in E16 mice, as well as in P24 and adult dunnarts. Interestingly, however, pan-Laminin is more broadly expressed throughout the parenchyma in the adult mouse brain and is associated with multiple axon tracts and thalamic nuclei, in addition to the leptomeninges and vasculature (Fig. 2a).

Comparison of the length of the IHF normalized to the total length of the telencephalic midline between E16 and adult mice revealed that the ratio of the IHF length to the total telencephalic midline length is significantly decreased by adulthood in mice (*p* = 0.0071, Mann-Whitney test; Fig. 2c). In contrast, the same comparison in dunnarts at the equivalent developmental stages (P24 and adult), revealed that the ratio of the IHF length to the total telencephalic midline length remains unchanged in dunnarts from early development into adulthood (*p* = 0.7000, Mann-Whitney test; Fig. 2b and c). These findings indicate that, in contrast to mouse development, interhemispheric remodeling does not occur during marsupial development.

**Fig. 3:**
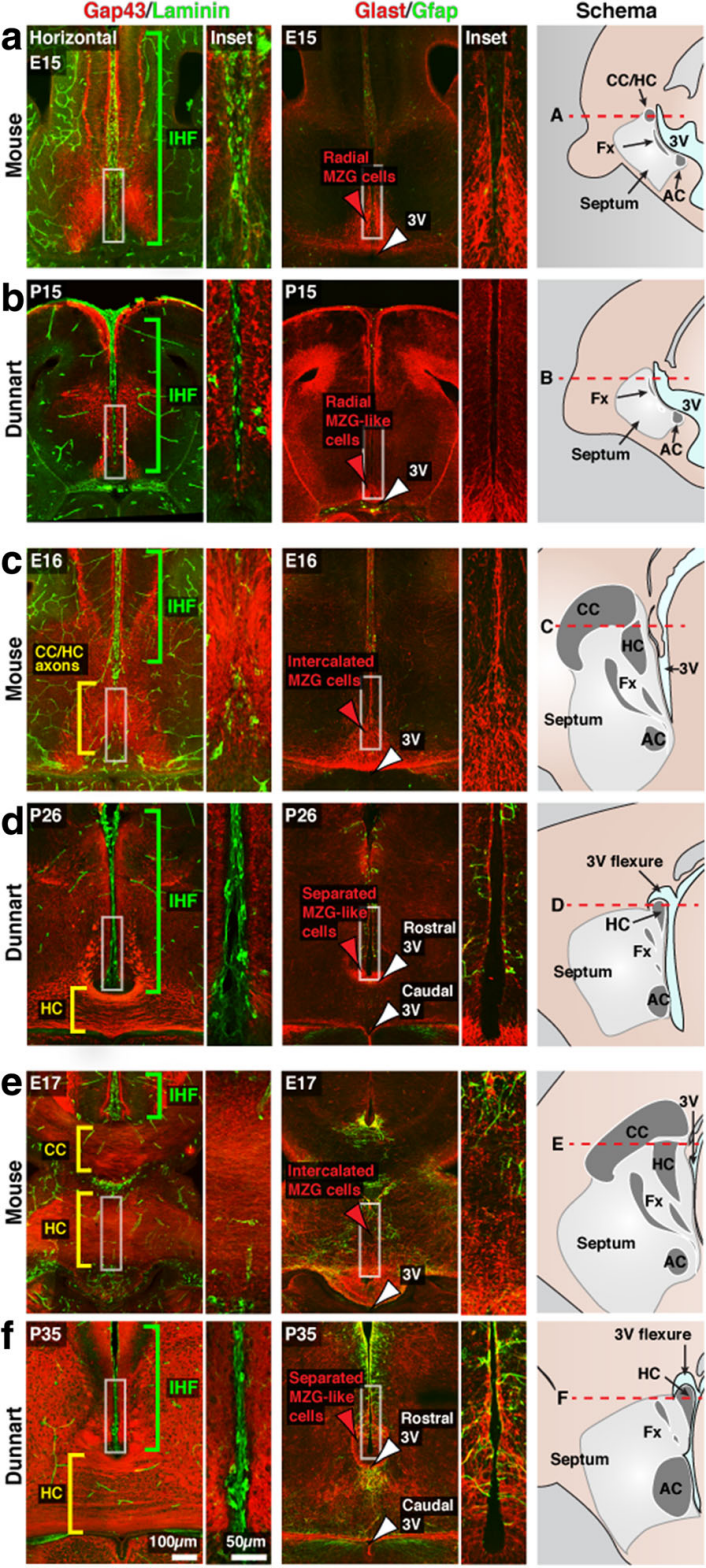
Astroglia do not initiate IHF remodeling in marsupials. Immunofluorescence was performed for Gap43 (*red*) and Laminin (*green*) to compare commissure formation and IHF development, and for Glast (*red*) and Gfap (*green*) to compare midline glial development in mice (**a**, **c**, and **e**) and dunnarts (**b**, **d**, and **f**) at equivalent developmental stages. The plane of each horizontal section (*red dotted* line) is represented by a mid-sagittal schematic on the right of each panel in **a** – **f**. Brackets indicate the distribution of the IHF (green) and commissural axons along the interhemispheric midline (*yellow*). Arrowheads indicate the distribution of MZG and MZG-like cells (*red arrowheads*) as well as the position of the third ventricle (3V; *white arrowheads*) along the interhemispheric midline. AC, anterior commissure; CC, corpus callosum; Fx, fornix; HC, hippocampal commissure

To better understand the differences in interhemispheric midline development between eutherian and non-eutherian mammals, we then compared the cellular development of this region between mice and dunnarts. In mice, maturation of radial MZG cells into multipolar MZG cells enables intercalation of these cells across the interhemispheric fissure, and triggers the subsequent remodeling of this structure [8]. This maturation is characterized by the molecular and morphological transition of Glast-positive radial cells into Gfap-positive multipolar cells [8]. To determine whether the absence of interhemispheric remodeling in marsupials may be related to the molecular and morphological maturation of MZG cells, we compared these processes across similar developmental stages in mice and dunnarts using immunohistochemistry. Specifically, labeling was performed for Laminin to delineate the extent of the IHF, and the axonal marker Gap43 to label commissural axons crossing the midline, while Glast and Gfap labeling was performed to assess the presence and maturation state of midline glia (Fig. 3).

In mice, interhemispheric remodeling and commissure formation largely occur between E15 and E17 (Fig. 3a–e). By experimental comparison of interhemispheric midline development between the two species, we found that P15, P26 and P35 (Fig. 3b–f) in dunnarts resemble E15, E16 and E17 in mice (Fig. 3a, c and e). During early stages of development, when commissure formation has not yet occurred (E15/P15), the interhemispheric midline appears almost identical in mice and dunnarts (Fig. 3a and b). In mice, radial MZG cells are Glast-positive/Gfap-negative bipolar cells that have an apical attachment to the surface of the third ventricle (3V) and a pial attachment to the IHF (Fig. 3a). At P15 in dunnarts, we observed an almost identical population that is Glast-positive/Gfap-negative and attached to both the 3V and the IHF (Fig. 3b). Thus, a radial MZG-like population is present by P15 in dunnarts, similar to that observed in mice at E15. However, from E16/P26 onwards, we identified several differences in interhemispheric midline development between mice and dunnarts (Fig. 3c–f). Notably, the caudal part of the septum begins to thicken dorsally in dunnarts by P26 (Fig. 3d), resulting in a distinct flexure of the 3V, which remains present at P35 (Fig. 3f). This flexure of the 3V is evident from horizontal sectioning of this region, which reveals both a rostral ventricular lumen that forms a small potential space adjacent to the base of the IHF (rostral 3V; Fig. 3d and f), and a second larger lumen caudal to the septum (caudal 3V; Fig. 3d and f) similar to that observed in mice (3V; Fig. 3a, c and e). Thus, via their attachment to the IHF, radial MZG-like cells become confined to the apical surface of the rostral 3V lumen in older dunnarts, but are not associated with the caudal 3V lumen, as are mouse MZG cells are at equivalent stages (compare Fig. 3c and e with d and f). Furthermore, we observed that this small MZG-like cell population in dunnarts does not expand, become multipolar or intercalate across the IHF, as mouse MZG cells do during commissure formation (compare Fig. 3c and e with d and f). Consistent with this, dunnart MZG-like cells, which maintain their radial attachment to the IHF, remain predominately Gfap-negative. In contrast, mouse MZG cells at equivalent stages substantially upregulate Gfap expression (compare Fig. 3c and e with d and f). This indicates that dunnart MZG-like cells maintain a more progenitor-like state during commissure formation, suggesting that the absence of interhemispheric remodeling in non-eutherian mammals is likely due to the lack of MZG-like cell differentiation. Subsequent intercalation of MZG cells therefore does not occur, resulting in retention of the IHF.

In the mouse, the hippocampal commissure crosses rostral to the entire 3V, through the remodeled region previously occupied by the IHF (HC; Fig. 3c and e). In contrast to this, we observed that the dunnart hippocampal commissure does not cross the midline through a region previously occupied by the IHF. Instead, axons of the hippocampal commissure in dunnarts cross caudal to the rostral lumen of the 3V, through the expanded caudal septum (Fig. 3d and f), which is consistent with previous observations of the hippocampal commissure in other marsupial species [14–16]. These results indicate that remodeling of the IHF is not required for the formation of the hippocampal commissure in marsupials, further suggesting that the cellular events mediating developmental remodeling of the IHF evolved exclusively in the ancestors of modern eutherians.

## Discussion

Early neuroanatomists identified the corpus callosum as a unique feature of the eutherian brain [15, 17–22]. However, the emergence of this commissure in eutherian ancestors has remained poorly understood. Here, we present comparative analyses of eutherian and non-eutherian brain development that highlight the evolutionary role of astroglial-mediated interhemispheric remodeling in the origin of the corpus callosum.

Our results indicate that interhemispheric remodeling does not occur in naturally acallosal monotremes and marsupials, such that the septum remains largely separated by the IHF throughout ontogeny in these lineages. Moreover, our histological observations from marsupials suggest that interhemispheric remodeling does not occur in these mammals due to an absence of MZG cell differentiation and intercalation. These results suggest a scenario whereby the corpus callosum co-evolved together with the cellular events mediating developmental remodeling of the IHF in eutherian mammals.

In both mice and humans, aberrant retention of the IHF, following defects in interhemispheric remodeling, forms a barrier that precludes callosal axons from crossing the midline [8]. Although it is not clear whether the leptomeninges and basement membrane within the eutherian IHF form a physical barrier to extending commissural axons, previously it has been suggested that Bmp7 expressed by the leptomeninges acts a repulsive cue for callosal axons in mice [23]. Whether the leptomeninges and basement membrane of non-eutherian mammals influence commissural axons in a similar manner to mice, however, is yet to be elucidated. In any case, our observations indicate that while the hippocampal commissure of eutherians may require interhemispheric remodeling and elimination of the IHF, similar to the corpus callosum, the formation of the marsupial hippocampal commissure does not require interhemispheric remodeling. Specifically, we found that while the hippocampal commissure in marsupials crosses through an expansion of the caudal septum, the eutherian hippocampal commissure crosses the midline through a region previously occupied by the IHF. These findings indicate that, together with the advent of interhemispheric remodeling, hippocampal commissural axons in eutherians adopted a new substrate through which to cross the midline. Thus, collectively, our observations suggest that interhemispheric remodeling likely provided a substrate for increasing numbers of commissural axons to cross the midline through a more rostral territory, otherwise occupied by non-permissive leptomeningeal tissue, in the ancestors of modern eutherians.

## Methods

### Animals

Wildtype CD1 and C57Bl/6 mice, as well as fat-tailed dunnarts, were bred at The University of Queensland. All breeding and experiments were performed with approval from The University of Queensland Animal Ethics Committee. Timed-pregnant mouse females were obtained by placing male and female mice together overnight, and the following morning was designated as embryonic day (E) 0 if a vaginal plug was detected. Male and female fat-tailed dunnarts (*Sminthopsis crassicaudata*) were housed together in mating groups, and females were pouch-checked every 3 days to determine postnatal day (P)0. Fixed platypus brains were loaned for imaging from the collection of Prof. John Nelson held at the Australian National Wildlife Collection, Commonwealth Scientific and Industrial Research Organisation (CSIRO).

### Ex-vivo magnetic resonance imaging

Three fixed whole adult C57Bl/6 brains, three fixed whole dunnart brains and two fixed whole platypus brains were incubated for 4 days in PBS containing 0.2% gadopentetate dimeglumine (Magnevist®), followed by image acquisition in Fomblin (Solvay Solexis). MR images were acquired for all brains using a 16.4 T scanner equipped with Micro2.5 imaging gradient and a 15 mm linear, surface acoustic wave coil (M2 M Imaging). FLASH (fast low angle shot, Paravision 5.1) images were acquired for mouse, dunnart and platypus brains with the following parameters: TR/TE = 50/12 ms, number of averages = 2 (dunnart and C57Bl/6) or 1 (platypus), field of view = 18.99 × 11.16 × 8 mm, acquisition matrix = 250 × 633, flip angle = 30°, at 30 × 30 × 30 μm isotropic resolution. Dicoms were converted and visualized using Mrtrix3 [24] (http://www.mrtrix.org) and itk-SNAP [25] (http://www.itksnap.org).

### Immunohistochemistry

Brain sections were processed for standard fluorescence immunohistochemistry as previously described [26]. Prior to incubation with primary antibodies, all sections were post-fixed in 4% paraformaldehyde and then subjected to antigen retrieval (125 °C for 4 min at 15 psi in sodium citrate buffer). Primary antibodies used were mouse anti-Gap43 (1:500; AB1987, Millipore), mouse anti-Gfap (1:500; MAB3402, Millipore), rabbit anti-Glast (or Eaat1; 1:250; ab416, Abcam), and chicken anti-Laminin (1:500; LS-C96142, LSBio). Alexa Fluor IgG (Invitrogen) and biotinylated-conjugated (Jackson Laboratories) secondary antibodies used in conjunction with Alexa Fluor 647-conjugated Streptavidin (Invitrogen) amplification, were all used according to the manufacturers’ instructions. Sections were counterstained to label cell nuclei using DAPI (Invitrogen) and coverslipped using ProLong Gold anti-fade reagent (Invitrogen) as mounting media.

### Image acquisition

Wide-field fluorescence imaging was performed with a Zeiss upright Axio-Imager Z1 microscope fitted with Axio- Cam HRc and HRm cameras, and images were acquired with Zen software (Carl Zeiss). Confocal images were acquired as multiple image projections of ~15 μm thick z-stacks using either an inverted Zeiss Axio-Observer fitted with a W1 Yokogawa spinning disk module, Hamamatsu Flash4.0 sCMOS camera and Slidebook 5.5 software or an inverted Nikon TiE fitted with a Spectral Applied Research Diskovery spinning disk module, Hamamatsu Flash4.0 sCMOS camera and Nikon NIS software. Images were pseudocolored to permit overlay, and then were cropped, sized, and contrast-brightness enhanced for presentation with Adobe Photoshop software.

### IHF measurements

Measurements of the IHF were performed using ImageJ software (NIH) on matched horizontal sections from the middle region of the telencephalon which were stained with DAPI (Invitrogen). To account for inter-individual brain size variability, the length of the IHF was then normalized to the entire rostro-caudal length of the telencephalon along the interhemispheric midline.

### Statistical analyses

Data were first assessed for normality of distribution with a D’Agostino-Pearson omnibus normality test, and then statistical differences between two groups were determined with a non-parametric Mann-Whitney test in Prism 6 software. *P* ≤ 0.05 was considered significant. All values are presented as mean ± standard error of the mean.

## Abbreviations

3V: Third ventricle
E: Embryonic day
IHF: Interhemispheric fissure
MZG: Midline zipper glia
P: Postnatal day
